# Changing Diet Quality in China during 2004–2011

**DOI:** 10.3390/ijerph14010013

**Published:** 2016-12-24

**Authors:** Yingying Huang, Hui Wang, Xu Tian

**Affiliations:** 1 College of Economics and Management, China Center for Food Security Studies, Nanjing Agricultural University, Nanjing 210095, Jiangsu, China; huangyingying623@126.com; 2 Department of Epidemiology, School of Public Health, Nanjing Medical University, Nanjing 211166, Jiangsu, China; huiwang@njmu.edu.cn

**Keywords:** diet quality index, nutrition transition, China, adults

## Abstract

Currently, under- and over-nutrition problems co-exist in China. However, systematic studies on the diet quality of Chinese residents have been scant. This study described the trend in diet quality of Chinese residents over a recent eight-year period and investigated the relevant influential factors. The data of Chinese adults aged 20–59 years was extracted from 2004, 2006, 2009, and 2011 China Health and Nutrition Survey. The China diet quality index (DQI) was employed to assess the diet quality of Chinese adults. The dietary consumption data of each individual was collected using a 24-h dietary recall and weighed food records implemented for three consecutive days. A mixed ordinary least squares regression model was applied to analyze the factors influencing the DQI scores of Chinese residents. Results showed that the diet quality of Chinese residents increased from 2004 to 2006, followed by a decrease in 2009 and 2011. The income, urbanicity index, and southern dummy were positively associated with DQI scores, whereas the size of household and labor intensity were negative predictors of DQI scores. The DQI scores also varied over BMI values. With an increase of the average income level in the future, the diet quality of Chinese residents is estimated to further improve. Moreover, urbanization could also contribute to reaching a more balanced diet.

## 1. Introduction

The nutrition transition has occurred among low–middle income countries, and scholars have begun shifting their research from under-nutrition to over-nutrition-related chronic disease, such as overweight and diabetes [1,2,3,4,5,6]. Diet quality plays a critical role in the progress of numerous chronic diseases, such as diabetes, hypertension, cardiovascular diseases and certain types of cancer [7,8,9,10,11,12,13,14,15,16,17]. Previous studies have indicated that the prevalence rate of obesity and nutrition-related chronic diseases in developing countries are rising [18,19,20], and China is no exception [18,20,21,22,23,24,25,26,27]. Therefore, research into diet quality warrants attention. After decades of rapid economic growth, China’s national income per capita has increased substantially, and the food consumption patterns in China have changed considerably [28]. The most evident related change is that the diet structure of Chinese residents has gradually shifted from the traditional diet of consuming diverse staple foods and vegetables to a diet containing a high fat content [29,30]. This alternation in diet structure changes diet quality inevitably and consequentially [31].

Few studies have revealed the developments of and changes in the diet quality of Chinese residents. One study [7] adopted the diet quality index-international (DQI-I) to analyze the differences and similarities between the diet quality in China and the United States. However, the DQI-I is not suitable for researching the diet quality of one country. Another study [32] employed the index of the desirable dietary pattern (DDP) score per capita to analyze the diet quality of Chinese residents, but only focused on rural people and cannot reveal the diet quality of all of China. Other studies have researched the diet quality of Chinese residents [17,20,33,34,35,36]; however, these studies have either only explored a certain aspect of diet quality (e.g., dietary patterns), recruited a certain group of people (e.g., children or elderly adults) as the research participants, or focused on the relationship between diet quality and a certain type of disease.

Stookey et al. [37] constructed an authoritative and effective index called the China diet quality index (DQI), which was specifically designed for measuring diet quality of residents in China. A DQI score converging to 0 indicates a more balanced diet. One advantage of the DQI is that it not only measures the nutrition transition, but can also be used to determine whether micronutrient requirements are being met. However, scholars have not yet to perform an in-depth analysis of the diet quality of Chinese residents with the DQI. Researching relevant topics can assist decision-makers with implementing correct food safety policies and nutrition and health policies. Therefore, the present study employed the China DQI to analyze the diet quality of Chinese residents over an eight-year period and its changes regarding various aspects, and subsequently applied a mixed ordinary least squares regression model to analyze the factors influencing the DQI scores of Chinese residents.

## 2. Materials and Methods

### 2.1. Study Subjects

The research data were obtained from the recent four waves (2004, 2006, 2009, and 2011) of the China Health and Nutrition Survey (CHNS). The CHNS is an international collaborative project between the Carolina Population Center at the University of North Carolina at Chapel Hill and the National Institute for Nutrition and Health at the Chinese Center for Disease Control and Prevention. The CHNS is an ongoing cohort survey on approximately 4000 families each year with the range of surveys covering the urban and rural areas in the following nine provinces (regions) before 2011: Guangxi, Guizhou, Henan, Heilongjiang, Hubei, Hunan, Jiangsu, Liaoning, and Shandong. The three municipalities of Beijing, Chongqing, and Shanghai were included in 2011. The content of the survey comprised the socioeconomic conditions, health services, residents’ diet structures, and their nutritional statuses. More details of the CHNS data can be found elsewhere [38].

A total of 46,830 observed values were obtained through an integration of age and food consumption information. Our study focused on Chinese adults aged 20–59 years. Respondents younger than 20 years or older than 59 years were excluded (*n* = 18,060). Therefore, a total of 28,770 observed measurements were collected. In addition, observations with incomplete personal information, family information and urbanization index (*n* = 12,264) were censored. We further removed 44 observations with unreliable energy intake (lower than 520 kcal/d or greater than 8000 kcal/d, sodium intake greater than 100 g/d). Sick persons (*n* = 2629) during the three-day survey period was also removed because their diet intake cannot be compared with healthy people directly. In total, 13,833 participants with complete information were included in the present study. (we have data from 3083, 3069, 3319, and 4362 individuals from 2004, 2006, 2009, and 2011, respectively).

### 2.2. Assessment of Food Consumption and Nutrient Intake

The CHNS collected details on individual food consumption (24-h recall) for three consecutive days within one week from all household members, including all food that they consumed at home and away from home. Data were recorded by trained interviewers through face-to-face interviews by using food models and pictures, and included the types, amounts, and locations of consumption of all food items consumed. The nutrient intake was converted to food consumption by using the China food composition table, which presents nutrient components for each food item [39,40]. Subsequently, each resident’s intake of the nutrients for three consecutive days was summed up and then was divided by three to obtain the average daily nutrient consumption for each individual. In addition, the CHNS also recorded food consumption for each household by measuring the household food inventory change, as well as the number of meals each household member ate at home. Detailed information about the survey has been previously reported [38].

### 2.3. Components, Cut-Off Values and Scoring of the DQI

For each individual, a DQI total score was calculated as the sum of the 10 DQI components, including diet variety, fruit and vegetables, protein, calcium, saturated fat, sodium, alcohol, energy, total carbohydrate, and total fat. The DQI components and scoring scheme are described in Table S1. The cut-off values and scoring frameworks for the 10 components were developed according to the Chinese Food Guide Pagoda, and Chinese and international dietary reference values [37]. A DQI score of 0 indicated a balanced diet, a DQI score of less than 0 indicated under-nutrition, and a DQI score of greater than 0 indicated over-nutrition. Components associated with under-nutrition included diet variety, fruits and vegetables, and protein; whereas saturated fat, sodium, and alcohol were components of over-nutrition. The remaining components contributed to both under and over-nutrition. The nutritional status was fully balanced when the value of each component was 0, individually.

#### 2.3.1. Diet Variety

Diet variety involved the variation of four major types of food: cereals and tubers; animal-based foods; beans and bean products; and vegetables and fruits. Each major type of food was then divided into 2–4 subgroups. Consumption of the different subgroups of these four major foods (Table S2) was the basis for calculating the diet variety score. A score of 1 for a subgroup was obtained if more than 25 g food of this subgroup was consumed. The major food type score was obtained by calculating the proportion of food subgroups within major food type. For example, if a major food type comprised four food subgroups, and the consumption of three of the food subgroups was more than 25 g, individually, then the total score for the food subgroups would be 3. Since the total number of food subgroups was 4 within this major food type, the score for this major food type would be 3/4. The range of values for the diet variety score was from −12 to 0. To calculate the diet variety score, we first need to set an intermediate variable m and then set the weight for the vegetables and fruits score in m to 40% and that for the three other types to 20%, respectively. Therefore, m can be calculated as follows:

m=Cereals and Tubers×20% + Animal Foods×20% + Beans and dairy products ×20% +Vegetables and fruit×40%

Subsequently, *m* was divided into 13 groups from the lowest to highest scores. The diet variety scores of the individuals from the lowest scoring group to the highest scoring group ranged from −12 to 0, increasing in increments of 1.

#### 2.3.2. Fruits and Vegetables

For the DQI, the fruits and vegetables score was obtained from the corresponding values derived by calculating the consumption of fruits and vegetables and carotene-rich vegetables (i.e., vegetables rich in carotene). The range of values for the fruits and vegetables score was from −12 to 0. To calculate the fruits and vegetables score, we first set an intermediate variable *n* and then recruited individuals with a daily intake of more than 2600 kcal:

n=6[(TVF/700)+(CV/300)]

and less than or equal to 2600 kcal:

n=6[(TVF/500)+(CV/200)]

where *TVF* are the total vegetables and fruits consumed (g) and *CV* are carotene-rich vegetables (g). Subsequently, *n* was divided into 13 groups from the lowest to highest scores. The fruits and vegetables scores of the individuals from the lowest scoring group to the highest scoring group ranged from−12 to 0, increasing in increments of 1.

#### 2.3.3. Total Energy, Calcium

To calculate the total energy and calcium score, we first referred the Chinese recommended nutrient intake (RNI) for energy and calcium [41] (Table S3). The total energy/calcium score was obtained by comparing the mean daily energy/calcium intake of the sampled individuals and RNI. Table S1 presents the value assignment methods.

#### 2.3.4. Total Carbohydrates, Total Fat, Saturated Fat, and Protein

On the basis of the conversion coefficients between carbohydrates and kcal energy (Table S1), the proportion of the energy provided by the mean daily intake of carbohydrates relative to the level of total energy was calculated. The total carbohydrate score was calculated by comparing this proportion with specific values, which was performed using the value assignment method presented in Table S1. Then the total fat, saturated fat and protein were calculated in the same way.

#### 2.3.5. Sodium and Alcohol

To calculate the sodium/alcohol score, the mean daily intake of sodium/alcohol by a sampled individual was compared with specific values. Table S1 presents the value assignment methods. Salt intake is not well recorded in the individual food consumption survey; we thus used the household food consumption survey data to calculate the sale and other ingredients intake for the whole family. The value was transferred into individual intake by dividing the total person-days (CHNS recorded the number of individuals ate at home for each meal, these data was then converted into person-day equivalents) of each family. In addition, to remove unreliable salt consumption data, we also delete observations with extremely high salt intake (greater than 100 g/d). The maximum limit of alcohol consumption is set to be 19 mL [37]. The score of alcohol component in DQI was calculated using pure alcohol consumption estimated from liquor and alcoholic beverage.

### 2.4. Co-Variates

To analyze the influential factors of the DQI score, the following variants were controlled in the multiple regression analysis: annual household income per capita, individual characteristic variables (age, gender, labor intensity, educational attainment, cigarette-smoking, BMI), family demographic variables (family size, number of children in the household, and number of older adults in the household), characteristic variables of the head of the household (age, gender, labor intensity, and education attainment), characteristic variables of the person who prepares the meals for the family (hereinafter referred to as the family meal provider) (age, labor intensity, and education attainment), dummy variables of regions (north or south), and urbanicity levels. Physical activity levels were measured according to the occupation type, and ranged from 1 to 5: namely, 1 = very light physical activity, working in a sitting position (for example, office worker or watch repairer); 2 = light physical activity, working in a standing position (for example, sales person or teacher); 3 = moderate physical activity (for example, student or driver); 4 = heavy physical activity (for example, farmer or dancer); and 5 = very heavy physical activity (for example, loader, logger, or miner). We further classified them into three groups: 1 and 2 are classified as light activity, 3 is taken as moderate activity, 4 and 5 are classified as heavy activity. Height and weight were measured directly, based on a standard protocol recommended by the World Health Organization, by trained health workers [42]. Body Mass Index (BMI) was divided into four categorical levels based on the criteria recommended by Working Group on Obesity in China [42], which are underweight: BMI < 18.5 kg·m^−2^; normal: BMI: 18.5–23.9 kg·m^−2^; overweight: BMI: 24.0–27.9 kg·m^−2^; general obesity: BMI ≥ 28.0 kg·m^−2^. Urbanicity level is defined by a multidimensional 12 component urbanization index, which captures population density, physical, social, cultural and economic environment, which has been explained in previous studies [42].

The annual household income per capita was deflated using the consumer price index of 2004.

### 2.5. Statistical Analysis

First, the three-day average DQI scores per person for the selected residents were calculated in each year, and the net change of each component was presented, which was further used to identify the contribution of a more balanced diet. To explore the diet quality of residents, their DQI scores were analyzed regarding the following six aspects: income, age, family size, BMI, labor intensity, and educational attainment. Finally, a multiple variable regression model was employed to explore the factors influencing the DQI scores of residents. The research data were unbalanced longitudinal data, and part observation data were obtained from the same participants or families in different waves; thus, the consumption structure and diet quality of these respondents or families were correlated. The cluster effects were controlled to eliminate the influences of such effects.

In this study, all data processing and analysis processes were completed using Stata/SE (version 11; Stata Corp, College Station, TX, USA). All statistical tests were two-tailed tests, and *p* < 0.05 was considered as statistically significant.

## 3. Results

### 3.1. Descriptive Analysis of Confounders

Table 1 presented the descriptive statistics (mean and standard deviations) of confounders according to different ranges of DQI scores. We found that higher DQI score is positively associated with some variables such as income, education, age, BMI, and urbanization index, but negatively associated with other variables such as children ratio, and family size.

### 3.2. Trend of DQI Scores and Its Components

Table 2 illustrates that the diet quality of Chinese residents increased from 2004 to 2006, but declined from 2006 to 2011. In general, the Chinese diet still remains on the under-nutrition part of the DQI spectrum. During the whole period, the total DQI score declined from −9.24 in 2004 to −13.78 in 2011 (*p* = 0.15). Although the scores of some components such as diet diversity, protein, total carbohydrate, and total fat increased (*p* = 0.01, 0.12, 0.03, and 0.01, respectively), the scores of other components such as fruit and vegetables, calcium, saturated fat, sodium, alcohol, and energy declined (*p* = 0.34, 0.02, 0.12, 0.06, 0.05, and 0.14, respectively). A simple contribution analysis on each component showed that diet diversity, protein, saturated fat, sodium, and alcohol contributed to a more balanced diet, while all other components diverged from a balanced diet during this period. Table S4 presented the changes of selected food items and nutrients which played a role in calculating the total DQI score.

### 3.3. Analysis of the DQI Scores of Chinese Residents Regarding Various Aspects

**Figure 1:**
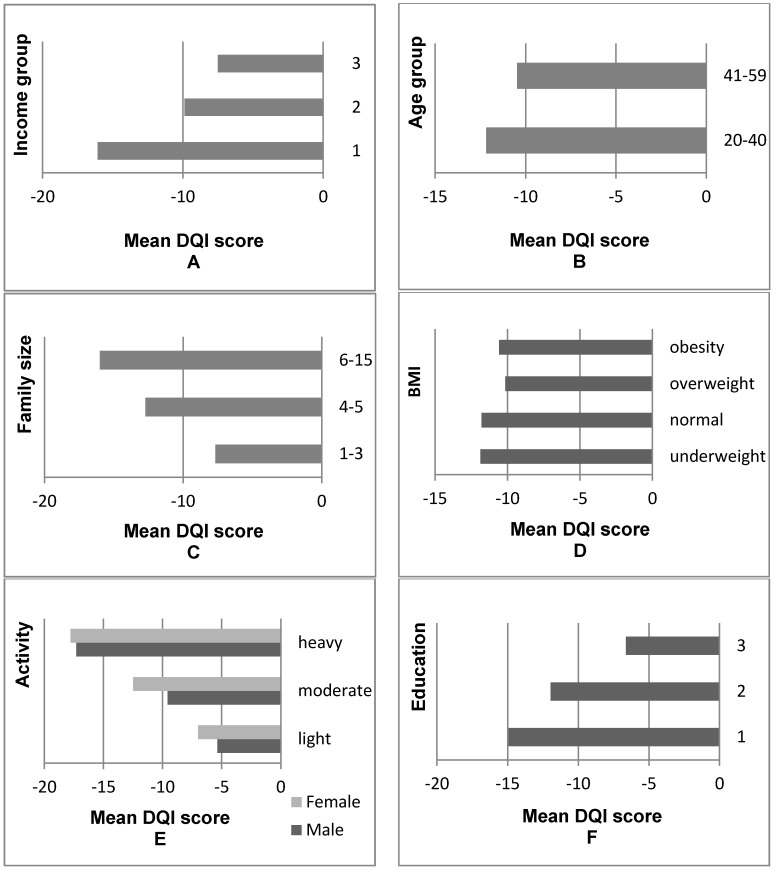
Comparison of DQI values across different groups. (**A**) 1: the poorest group; 2: middle income group; 3: the richest group; (**B**) Young adult: 20–40 years old, old adult: 41–59 years old; (**C**) Number of individuals in the family; (**D**) Underweight: Body Mass Index (BMI) < 18.5 kg·m^−2^; normal: 18.5 ≤ BMI ≤ 23.9 kg·m^−2^; overweight: 24.0 ≤ BMI ≤ 27.9 kg·m^−2^; general obesity: BMI ≥ 28.0 kg·m^−2^; (**E**) Light: very light and light physical activity; moderate: moderate physical activity; heavy: heavy and very heavy physical activity; (**F**) Education: 1 = primary education; 2 = secondary education; 3 = tertiary education.

Figure 1 illustrated that the diet quality of Chinese residents improved as their income levels increased. The DQI score per capita of the highest income group was −7.51, which was 53% higher than the DQI score per capita of the lowest income group (−16.07). However, the diet quality of the highest income group did not achieve the standard of a balanced diet. According to the age distribution, the mean diet quality of older adults (aged 41–59 years) was more satisfactory than that of younger residents (aged 20–40 years). The diet quality of residents from small families was more satisfactory than that of residents from large families. The DQI scores of overweight and obese people were higher than those of normal weight people. Moreover, Figure S1 also confirmed the inverse-U shape relationship between DQI scores and BMI. The diet quality of both men and women engaged in work requiring high labor intensity was unsatisfactory; in addition, the diet quality of men was relatively more satisfactory than that of women when the members of both groups were engaged in work requiring the same labor intensity. Residents with high educational attainment had more balanced DQI scores.

### 3.4. Analysis of the Association between Co-Variants and the DQI Scores

To further explore the factors influencing the diet quality of Chinese residents, a multiple variable regression analysis was performed and results are presented in Table 3. 

We found that poor people had significantly lower DQI scores than rich people (*p* < 0.001). Older adults (aged 40–60 years old) and people engaged in low-physical-activity jobs had higher DQI scores than their counterparts (all *p* < 0.001). Overweight and obese individuals had more balanced diets than people with normal weight (all *p* < 0.001). In addition, people living in large families or families with more children were associated with lower DQI scores (all *p* < 0.001). The DQI score was also influenced by characteristics of family meal providers, but not associated with characteristics of the household head. In particular, if the people who cooked during the survey period had lower physical activity levels, were well educated, and older, the DQI score of all family members was higher (*p* < 0.001; *p* = 0.035; and *p* = 0.010, respectively). Extremely significant regional differences were also observed for the diet quality of Chinese residents as follows: (1) the diet quality of residents living in southern regions was more satisfactory than that of residents living in northern regions (*p* < 0.001); and (2) people living in more urbanized areas had higher DQI scores (*p* < 0.001). Except for the aforementioned factors, the other factors presented in Table 3 did not exhibit significant influences on the DQI scores.

## 4. Discussion

By calculating the DQI scores, we explored the diet quality of Chinese residents over an eight-year period and its changes, and further analyzed the factors influencing the DQI scores. According to the inter-annual changes, the diet quality of Chinese residents improved from 2004 to 2006, followed by a decrease from 2006 to 2011; meanwhile, the current overall level of diet quality requires further improvement, a finding that was consistent with a previous study [32].

### 4.1. The Change of the Total DQI Score over the Eight-Year Period

We found that the total DQI score declined slightly from 2004 to 2011, indicating diverging from a balanced diet. Component decomposition found an increased consumption of animal-based foods and milk and dairy products. Since the primary source of fat and protein are animal-based foods and nuts, the intake of protein and total fat by Chinese residents thus increased. Therefore, the increased intake of protein and total fat, and the decreased intake of total carbohydrate, would improve the total DQI score. In spite of this, the consumption of fruit and vegetables and the intake of calcium, total energy, saturated fat, sodium, and alcohol, decreased a lot, resulting in the total DQI score declining in 2011. Similar findings have been presented in another current study [37]. Fortunately, the diet quality of Chinese residents is estimated to further improve in the future because of an increased consumption of animal-based foods [43] and a decreased intake of total carbohydrate. Studies conducted in other countries have also indicated that an increase of animal-based food consumption improves diet quality [6,44,45]. Current studies have indicated that a high intake of total energy lowers the diet quality of residents in most developed countries [6,44,45], and that an increased consumption of meats and dairy products and intake of total fat and saturated fat do not improve diet quality [6,12,44,45,46,47,48,49]. These phenomena are associated with the following aspects: First, China is a developing country and is in the process of nutrition transition; thus, a certain number of people are still affected by under-nutrition. An increased intake of total energy is beneficial for improving Chinese residents’ diet quality overall. This positive association was further confirmed using a nonparametric polynomial estimation (Figure S2). Second, the index system used for assessing the diet quality in developed countries is not identical to the DQI for China. The Chinese DQI explicitly defines diet quality considering both under- and over-nutrition. It also defines diet quality by incorporating information about the direction, in addition to the distance, strayed from an ideal diet quality [37]. The index system for diet quality assessment applied by developed countries focuses relatively more on the negative effects of over-consuming meat, processed meat, and dairy products [6]. The aforementioned results revealed that the main reasons for the 2011 decrease of the total DQI score of Chinese residents were associated with the following structural changes: a decrease of the consumption of fruit and vegetables and the intake of calcium, total energy, saturated fat, sodium, and alcohol.

### 4.2. The Factors Associated with DQI Scores

We determined that Chinese residents’ diet quality is associated with some evident characteristics. First, the diet quality of the higher income group was relatively more satisfactory. The regression analysis results further verified that the household net income per capita positively influenced the DQI scores, which is consistent with most current studies [6,32,37,50]. In general, income level represents economic strength and purchasing power, and directly influences the consumed food type by people and the nutrition structure of their diets [29,51,52]. Second, the diet quality of relatively older (41–59) Chinese residents was more satisfactory, consistent with the results found in Australians [1,6]. One possible reason is that older people care more about the healthy impacts of food consumption [12], and those who were retired from work had relatively more free time and the adequate financial capability to improve their diet quality. Third, families with a lower dependence ratio or small size had more balanced diets, which could be attributed to the lower economic burdens in these families [32]. Fourth, the diet quality of overweight and obese subjects was more satisfactory than normal weight people. Similar findings have been offered by other current studies [37]. In addition, the diet quality of residents engaged in work requiring high labor intensity was less satisfactory, which was different from the results indicated by some previous studies [3,6]. The main reason for this might be because Chinese residents, both men and women, who are engaged in work requiring high labor intensity are mostly engaged in manual labor jobs; thus, they do not have the sufficient financial capability to improve their diet quality and lack certain nutritional knowledge. Fifth, southerners and residents living in highly urbanized areas were more satisfactory than their counterparts [37]. This could be attributed to the higher food availability and more reasonable diet structure in southern and highly urbanized areas [7,34,53]. In addition, residents in highly urbanized areas had more diversified food and lower demand for high-calorie-density food due to their lifestyle and higher accessibility to food markets [54]. Furthermore, some other factors also significantly influenced diet quality; specifically, the educational attainment and age of a family meal provider were positively correlated with the family members’ diet quality. Contrarily, the labor intensity of a family meal provider was negatively correlated with the family members’ diet quality. Previous study [29] also verified that the characteristics of the family meal provider influenced the nutritional intake of family members.

### 4.3. Limitation of the Present Study

This study suffers from several limitations. First, we only used data after 2004 due to the inconsistence in the statistical approach and food codes implemented by the CHNS. Second, the mean of three-day food consumption data was used to proxy each person’s daily diet, which might be prone to a misclassification errors due to seasonal variation in food consumption. Fortunately, the CHNS data were collected in autumn, a period in which food availability differences are minimized [7], and the mean of the intake distribution is drawn from a large, representative sample of a group is not affected by day-to-day variation [49,55]. Finally, nutrient components of processed food might suffer from large measurement error due to mismatch between the limited number of processed foods in the China Food Composition Table and the rising consumption and variety of processed food in reality.

### 4.4. The Advantages of the Present Study

Our study provides a general picture of Chinese diet quality as it relates to the rapid nutrition transition in China in the past decade. Findings detected in this study can be employed to the whole of China regarding the representative distribution and large size of the CHNS sample. Moreover, the results offer insights that can be used for developing public health programs that encourage healthy diets, not only in China but also in other countries experiencing similar transitions of nutritional intake. In addition, the approach used in this study can be applied to similar data in other countries.

## 5. Conclusions

The total DQI score of Chinese residents is falling and 73% of Chinese residents still stayed in the under-nutrition spectrum. We found substantial differences in diet quality in different groups of individuals. We further indicated that income, BMI, family size, labor intensity, and the family dependency ratio influenced diet quality. Finally, major determinants of dietary change in China are economic status and urbanization [37]. Therefore, Chinese residents’ diet quality is estimated to improve as their income level rises and urbanization progresses in the future.

## Figures and Tables

**Table 1:** Descriptive characteristics of control variables.

Control Variables	DQI (<−20)		DQI (−20~−10)		DQI (−10~0)		DQI (>0)		Total	
	Mean	SD ^1^	Mean	SD ^1^	Mean	SD ^1^	Mean	SD ^1^	Mean	SD ^1^
Income	6648	9053	8581	10,930	10,155	11,998	10,335	12,606	8821	11,255
Activity level **^2^**	3.24	0.88	2.95	0.90	2.78	0.88	2.57	0.80	2.90	0.90
Education **^3^**	1.82	0.76	1.98	0.78	2.10	0.79	2.19	0.78	2.02	0.79
Age	42.19	10.66	42.75	10.33	43.03	10.35	43.15	10.05	42.75	10.37
Gender **^4^**										
Male (%)	13.66	0.00	10.42	0.00	9.87	0.00	12.95	0.00	46.90	0.00
Female (%)	15.57	0.00	12.29	0.00	11.18	0.00	14.06	0.00	53.10	0.00
BMI	23.38	3.56	23.51	3.69	23.61	3.98	23.73	3.89	23.55	3.77
Children ratio	0.20	0.18	0.19	0.17	0.18	0.18	0.16	0.17	0.18	0.17
Old people ratio	0.06	0.12	0.06	0.13	0.06	0.13	0.06	0.13	0.06	0.13
Family size	4.47	1.75	4.22	1.70	3.98	1.57	3.78	1.48	4.12	1.66
Householder status										
Activity level **^2^**	3.16	1.12	2.81	1.19	2.60	1.20	2.32	1.14	2.73	1.20
Education **^3^**	1.79	0.76	1.95	0.78	2.08	0.79	2.17	0.78	1.99	0.79
Age	45.98	8.87	46.13	8.69	46.47	8.66	46.32	8.38	46.21	8.65
Gender **^4^**										
Male (%)	19.96	0.00	15.30	0.00	14.77	0.00	18.46	0.00	68.49	0.00
Female (%)	9.27	0.00	7.42	0.00	6.28	0.00	8.54	0.00	31.51	0.00
Cook status										
Activity level **^2^**	3.04	1.09	2.68	1.14	2.45	1.12	2.19	1.07	2.61	1.15
Education **^3^**	1.64	0.73	1.83	0.78	1.95	0.80	2.04	0.81	1.86	0.79
Age	43.70	8.50	44.25	8.33	44.70	8.08	44.89	7.96	44.36	8.24
Currently smoking **^2^**										
Yes (%)	8.15	0.00	6.49	0.00	6.04	0.00	7.66	0.00	28.35	0.00
No (%)	21.07	0.00	16.22	0.00	15.01	0.00	19.35	0.00	71.65	0.00
Region **^4^**										
South (%)	13.06	0.00	12.67	0.00	12.56	0.00	16.56	0.00	54.85	0.00
North (%)	16.17	0.00	10.05	0.00	8.49	0.00	10.45	0.00	45.15	0.00
Urbanization index **^5^**	59.20	19.15	65.56	19.71	69.82	19.57	74.26	18.27	66.95	20.01
Number of observations	4043		3142		2912		3736		13,833	

**^1^** SD: standard deviation; **^2^** Physical activity levels (1 = very light physical activity, working in asitting position; 2 = lightphysical activity, working in a standing position; 3 = moderate physical activity; 4 = heavy physical activity; and 5 = veryheavy physical activity); **^3^** Education (1 = primary education; 2 = secondary education; 3 = tertiary education); **^4^** Mean is measured in percentage; **^5^** Defined by a multidimensional 12-component urbanization index, which captures the population density, physical, social, cultural and economic environment.

**Table 2:** Changing diet quality index (DQI) scores and its components.

Role	Components	2004 (*n* = 3083)		2006 (*n* = 3069)		2009 (*n* = 3319)		2011 (*n* = 4362)		*p* ^2^	Net Change ^3^	Contribution ^4^
		Mean	SD ^1^	Mean	SD ^1^	Mean	SD ^1^	Mean	SD ^1^			
	Total DQI score	−9.24	19.31	−7.22	18.65	−13.13	15.88	−13.78	15.00	0.15	−4.54	−
under-nutrition	Diet variety	−6.84	3.69	−6.42	3.79	−5.60	3.64	−5.42	3.68	0.01	1.42	+
	Fruit and vegetables	−5.74	3.74	−5.49	3.70	−5.53	3.60	−6.90	3.72	0.34	−1.16	−
	Protein	−0.51	1.85	−0.53	1.89	−0.46	1.84	−0.34	1.61	0.12	0.17	+
	Calcium	−7.88	3.56	−8.10	3.39	−8.42	3.16	−8.93	2.71	0.02	−1.05	−
over-nutrition	Saturated fat	0.59	2.20	0.73	2.44	0.04	0.57	0.02	0.43	0.12	−0.57	+
	Sodium	7.44	3.69	6.91	3.78	0.20	1.17	0.15	1.00	0.06	−7.29	+
	Alcohol	0.39	1.40	0.39	1.42	0.32	1.24	0.31	1.25	0.05	−0.08	+
under- and over-nutrition	Energy	−1.78	5.66	−1.91	5.63	−2.12	5.60	−3.40	5.77	0.14	−1.62	−
	Total carbohydrate	1.29	5.77	2.33	5.84	2.92	5.26	4.49	5.41	0.03	3.20	−
	Total fat	3.79	5.97	4.86	5.77	5.53	5.23	6.24	5.04	0.01	2.45	−

**^1^** SD is the abbreviation of standard deviation; **^2^** *p* value of trend test; **^3^** Net change between 2011 and 2004; **^4^** “−” refers to contributing negatively to a more balanced diet (DQI score = 0), while “+” refers to contributing positively to a more balanced diet (DQI score = 0).

**Table 3:** Association between DQI scores and control variables as determined by multivariate ordinary least squares regression (*n* =13,833).

Control Variables	Coefficient	95% CI	*p*
Income **^1^**			
Middle income	4.816	(3.966, 5.666)	<0.001
High income	5.316	(4.327, 6.304)	<0.001
Activity level **^2^**	−1.891	(−2.478, −1.304)	<0.001
Education **^3^**	0.257	(−0.300, 0.814)	0.366
Age **^4^**	1.318	(0.595, 2.042)	<0.001
Gender **^5^**	0.187	(−0.469, 0.843)	0.576
BMI **^6^**			
Underweight	−0.125	(−1.505, 1.256)	0.859
Overweight	1.513	(0.892, 2.134)	<0.001
Obesity	1.883	(0.962, 2.805)	<0.001
Children ratio	−2.631	(−4.913, −0.349)	0.024
Old people ratio	−2.609	(−5.329, 0.111)	0.060
Family size **^7^**			
4–5	−2.128	(−2.935, −1.321)	<0.001
6–15	−3.389	(−4.504, −2.274)	<0.001
Householder status			
Activity level **^2^**	0.041	(−0.719, 0.801)	0.916
Education **^3^**	0.469	(−0.238, 1.176)	0.193
Age **^4^**	−0.052	(−0.112, 0.009)	0.095
Gender **^5^**	0.151	(−0.626, 0.927)	0.703
Cook status			
Activity level **^2^**	−1.602	(−2.352, −0.852)	<0.001
Education **^3^**	0.701	(0.050, 1.352)	0.035
Age **^4^**	0.079	(0.019, 0.139)	0.010
Currently smoking **^8^**	0.580	(−0.208, 1.368)	0.149
Year **^9^**	−1.606	(−1.741, −1.471)	<0.001
Region **^10^**	5.200	(4.450, 5.949)	<0.001
Urbanization index **^11^**	0.097	(0.071, 0.123)	<0.001
constant	−14.863	(−18.715, −11.011)	<0.001

**^1^** Household net income per capita is deflated to 2004 price, and low income group is taken as the reference category; **^2^** 1 = light, 2 = moderate, 3 = heavy; **^3^** Highest education completed, 1 = primary education, 2 = secondary education, 3 = tertiary education; **^4^** 0: young adults (20–40 years old), 1: old adults (41–60 years old); **^5^** 1 = Male, 0 = Female; **^6^** Underweight: BMI < 18.5 kg·m^−2^, normal: 18.5 ≤ BMI ≤ 23.9 kg·m^−2^ (reference group), overweight: 24.0 ≤ BMI ≤ 27.9 kg·m^−2^, general obesity: BMI ≥ 28.0 kg·m^−2^; **^7^** Reference group: 1–3 members; **^8^** Smoking status: 1 = Yes, 0 = No; **^9^** 0 = 2004, 2 = 2006, 5 = 2009, 7 = 2011; **^10^** Region: 1 = South (Jiangsu, Hunan, Hubei, Guangxi, Guizhou, Shanghai, Chongqing), 0 = North (Liaoning, Shandong, Henan, Heilongjiang, Beijing); **^11^** Defined by a multidimensional 12-component urbanization index, which captures the population density, physical, social, cultural and economic environment.

## Supplementary Materials

The following are available online at www.mdpi.com/1660-4601/14/1/13/s1. Figure S1: Association between DQI and BMI. 1. Solid line refers to fitted value, and the two dashed lines refer to the 95% confidence interval; 2. Lower and upper 5% values are censored to remove outliers, Figure S2: Association between DQI and total energy. 1. Solid line refers to fitted value, and the two dashed lines refer to the 95% confidence interval; 2. Lower and upper 5% values are censored to remove outliers, Table S1: Components of the Chinese DQI applied to the data, Table S2: Diet variety food subgroups, Table S3: Chinese RNI for energy and calcium, Table S4: Mean intake of selected foods and nutrients by DQI total score.

## References

[1] McNaughton S.A., Ball K., Crawford D., Mishra G.D. (2008). An index of diet and eating patterns is a valid measure of diet quality in an Australian population. J. Nutr..

[2] Collins C.E., Young A.F., Hodge A. (2008). Diet quality is associated with higher nutrient intake and self-rated health in mid-aged women. J. Am. Coll. Nutr..

[3] Kaluza J., Hakansson N., Brzozowska A., Wolk A. (2009). Diet quality and mortality: A population-based prospective study of men. Eur. J. Clin. Nutr..

[4] Tucker K.L. (2010). Dietary patterns, approaches, and multicultural perspective. Appl. Physiol. Nutr. Metab..

[5] Verger E.O., Mariotti F., Holmes B.A., Paineau D., Huneau J.F. (2012). Evaluation of a diet quality index based on the probability of adequate nutrient intake (pandiet) using national French and U.S. dietary surveys. PLoS ONE.

[6] Zarrin R., Ibiebele T.I., Marks G.C. (2013). Development and validity assessment of a diet quality index for Australians. Asia Pac. J. Clin. Nutr..

[7] Kim S., Haines P.S., Siega-Riz A.M., Popkin B.M. (2003). The diet quality index-international (DQI-I) provides an effective tool for cross-national comparison of diet quality as illustrated by China and the United States. J. Nutr..

[8] Azadbakht L., Mirmiran P., Esmaillzadeh A., Azizi F. (2006). Dietary diversity score and cardiovascular risk factors in tehranian adults. Public Health Nutr..

[9] Salas-Salvadó J., Martinez-González M.Á., Bulló M., Ros E. (2011). The role of diet in the prevention of type 2 diabetes. Nutr. Metab. Cardiovasc. Dis..

[10] Hu F.B. (2011). Globalization of diabetes: The role of diet, lifestyle, and genes. Diabetes Care.

[11] Thomas T., Pfeiffer A.F.H. (2012). Foods for the prevention of diabetes: How do they work?. Diabetes/Metab. Res. Rev..

[12] Gao Z., Yu X., Lee J.-Y. (2013). Consumer demand for diet quality: Evidence from the healthy eating index consumer demand for diet quality: Evidence from the healthy eating index. Aust. J. Agric. Resour. Econ..

[13] Flores G., Lin H. (2013). Factors predicting overweight in U.S. kindergartners. Am. J. Clin. Nutr..

[14] Gubbels J.S., van Assema P., Kremers S.P. (2013). Physical activity, sedentary behavior, and dietary patterns among children. Curr. Nutr. Rep..

[15] Roytio H., Jaakkola J., Hoppu U., Poussa T., Laitinen K. (2015). Development and evaluation of a stand-alone index for the assessment of small children’s diet quality. Public Health Nutr..

[16] Zhang J., Wang H., Wang Y., Xue H., Wang Z., Du W., Su C., Zhang J., Jiang H., Zhai F. (2015). Dietary patterns and their associations with childhood obesity in China. Br. J. Nutr..

[17] Batis C., Mendez M.A., Gordon-Larsen P., Sotres-Alvarez D., Adair L., Popkin B. (2016). Using both principal component analysis and reduced rank regression to study dietary patterns and diabetes in Chinese adults. Public Health Nutr..

[18] Yang G., Kong L., Zhao W., Wan X., Zhai Y., Chen L.C., Koplan J.P. (2008). Emergence of chronic non-communicable diseases in China. Lancet.

[19] Whiting D.R., Guariguata L., Weil C., Shaw J. (2011). Idf diabetes atlas: Global estimates of the prevalence of diabetes for 2011 and 2030. Diabetes Res. Clin. Pract..

[20] Hong X., Xu F., Wang Z., Liang Y., Li J. (2016). Dietary patterns and the incidence of hyperglyacemia in China. Public Health Nutr..

[21] Wang H., Du S., Zhai F., Popkin B.M. (2007). Trends in the distribution of body mass index among Chinese adults, aged 20–45 years (1989–2000). Int. J. Obes. (2005).

[22] Dearth-Wesley T., Wang H., Popkin B.M. (2008). Under- and overnutrition dynamics in Chinese children and adults (1991–2004). Eur. J. Clin. Nutr..

[23] Wildman R.P., Gu D., Muntner P., Wu X., Reynolds K., Duan X., Chen C.S., Huang G., Bazzano L.A., He J. (2008). Trends in overweight and obesity in Chinese adults: Between 1991 and 1999–2000. Obesity.

[24] National Center for Chronic and Non-Communicable Disease Control and Prevention (2010). Report on Chronic Disease Risk Factor Surveillance in China.

[25] Xi B., Liang Y., He T., Reilly K.H., Hu Y., Wang Q., Yan Y., Mi J. (2012). Secular trends in the prevalence of general and abdominal obesity among Chinese adults, 1993–2009. Obes. Rev..

[26] Li M.Z., Su L., Liang B.Y., Tan J.J., Chen Q., Long J.X., Xie J.J., Wu G.L., Yan Y., Guo X.J. (2013). Trends in prevalence, awareness, treatment, and control of diabetes mellitus in mainland China from 1979 to 2012. Int. J. Endocrinol..

[27] Zuo H., Shi Z., Hussain A. (2014). Prevalence, trends and risk factors for the diabetes epidemic in China: A systematic review and meta-analysis. Diabetes Res. Clin. Pract..

[28] Zheng Z., Gao Y., Zhao Y. (2015). The effect of income growth on urban food consumption patterns. China Econ. Q..

[29] Tian X., Yu X. (2015). Using semiparametric models to study nutrition improvement and dietary change with different indices: The case of China. Food Policy.

[30] Tian X., Yu X. (2013). The demand for nutrients in China. Front. Econ. China.

[31] Du S., Mroz T.A., Zhai F., Popkin B.M. (2004). Rapid income growth adversely affects diet quality in China—Particularly for the poor!. Soc. Sci. Med. (1982).

[32] Xiao H., Wang Z. (2008). Rural households dietary quality status and its influence factors analysis in Chinese poor areas. Chin. Rural Econ..

[33] Zeng F.F., Xue W.Q., Cao W.T., Wu B.H., Xie H.L., Fan F., Zhu H.L., Chen Y.M. (2014). Diet-quality scores and risk of hip fractures in elderly urban Chinese in Guangdong, China: A case-control study. Osteoporos. Int..

[34] Liu J., Shively G.E., Binkley J.K. (2014). Access to variety contributes to dietary diversity in China. Food Policy.

[35] Cheng G., Duan R., Kranz S., Libuda L., Zhang L. (2016). Development of a dietary index to assess overall diet quality for Chinese school-aged children: The Chinese children dietary index. J. Acad. Nutr. Diet..

[36] Chan R., Leung J., Woo J. (2016). A prospective cohort study to examine the association between dietary patterns and sarcopenia in Chinese community-dwelling older people in Hong Kong. J. Am. Med. Dir. Assoc..

[37] Stookey J.D., Wang Y., Ge K., Lin H., Popkin B.M. (2000). Measuring diet quality in China: The INFH-UNC-CH diet quality index. Eur. J. Clin. Nutr..

[38] Zhang B., Zhai F.Y., Du S.F., Popkin B.M. (2014). The China health and nutrition survey, 1989–2011. Obes. Rev..

[39] Institute of Nutrition and Food Safety (2004). China Food Composition 2004.

[40] Institute of Nutrition and Food Safety (2002). China Food Composition 2002.

[41] Institute of Nutrition and Food Safety (2009). China Food Composition 2009.

[42] Xu X., Hall J., Byles J., Shi Z. (2015). Assessing dietary quality of older Chinese people using the Chinese Diet Balance Index (DBI). PLoS ONE.

[43] Wang Z.H., Zhai F.Y., Wang H.J., Zhang J.G., Du W.W., Su C., Zhang J., Jiang H.R., Zhang B. (2015). Secular trends in meat and seafood consumption patterns among Chinese adults, 1991–2011. Eur. J. Clin. Nutr..

[44] Trichopoulou A., Costacou T., Bamia C., Trichopoulos D. (2003). Adherence to a mediterranean diet and survival in a greek population. N. Engl. J. Med..

[45] Fung T.T., Rexrode K.M., Mantzoros C.S., Manson J.E., Willett W.C., Hu F.B. (2009). Mediterranean diet and incidence of and mortality from coronary heart disease and stroke in women. Circulation.

[46] Scali J., Richard A., Gerber M. (2001). Diet profiles in a population sample from Mediterranean southern France. Public Health Nutr..

[47] Bodnar L.M., Siega-Riz A.M. (2002). A diet quality index for pregnancy detects variation in diet and differences by sociodemographic factors. Public Health Nutr..

[48] Wong J.E., Parnell W.R., Howe A.S., Black K.E., Skidmore P.M. (2013). Development and validation of a food-based diet quality index for New Zealand adolescents. BMC Public Health.

[49] Fulgoni V.L., Chu Y., O’Shea M., Slavin J.L., DiRienzo M.A. (2015). Oatmeal consumption is associated with better diet quality and lower body mass index in adults: The national health and nutrition examination survey (NHANES), 2001–2010. Nutr. Res..

[50] Darmon N., Drewnowski A. (2008). Does social class predict diet quality?. Am. J. Clin. Nutr..

[51] De Z., Yu X. (2015). Calorie elasticities with income dynamics: Evidence from the literature. Appl. Econ. Perspect. Policy.

[52] Ha D.T.P., Feskens E.J., Deurenberg P., le Mai B., Khan N.C., Kok F.J. (2011). Nationwide shifts in the double burden of overweight and underweight in Vietnamese adults in 2000 and 2005: Two national nutrition surveys. BMC Public Health.

[53] Drewnowski A., Monsivais P., Maillot M., Darmon N. (2007). Low-energy-density diets are associated with higher diet quality and higher diet costs in French adults. J. Am. Diet. Assoc..

[54] Huang J. (1999). Social development, urbanization and food consumption. Soc. Sci. China.

[55] Guenther P.M., Kott P.S., Carriquiry A.L. (1997). Development of an approach for estimating usual nutrient intake distributions at the population level. J. Nutr..

