# Supplementary material for: Changing Diet Quality in China during 2004–2011
Source: Int J Environ Res Public Health. 2016 Dec 24;14(1):13. doi: 10.3390/ijerph14010013 (PMC5295264; doi:10.3390/ijerph14010013)
Supplement: Supplementary file 1 [file ijerph-14-00013-s001.pdf]

Supplementary Materials: Changing Diet Quality in China during 2004–2011

Yingying Huang, Hui Wang and Xu Tian

Table S1. Components of the Chinese DQI applied to the data.

Components	Score	Scoring Criteria	Energy Conversion Coefficient
Diet variety	−12–0	depends on group [1]	— —
Fruit and vegetables	−12–0	depends on group [2]	— —
Protein	−10–0	<8% of energy = −10<br>8%–9% of energy = −5<br>≥10% of energy = 0	4
Calcium	−10–0	<50%RNI = −10 [3]<br>50%–66%RNI = −5 [3]<br>≥67%RNI = 0 [3]	— —
Saturated fat	0–10	≤7% of energy = 0<br>8%–10% of energy = 5<br>>10% of energy = 10	9
Sodium	0–10	<2400 mg = 0<br>2400–4000 mg = 5<br>≥4000 mg = 10	— —
Alcohol	0–6	<50% of maximum = 0<br>51%–100% of maximum = 3<br>≥100% of maximum = 6	— —
Energy	−10–10	<70%RNI = −10<br>70%–79%RNI = −5<br>80%–119%RNI = 0<br>120%–129%RNI = 5<br>≥130%RNI = 10	— —
Total carbohydrate	−10–10	<50% of energy = 10<br>50%–54% of energy = 5<br>55%–69% of energy = 0<br>70%–74% of energy = −5<br>≥75% of energy = −10	4
Total fat	−10–10	<10% of energy = −10<br>10%–14% of energy = −5<br>15%–24% of energy = 0<br>25%–29% of energy = 5<br>≥30% of energy = 10	9

Source: Stookey et al. [1] and Institute of Nutrition and Food Safety [2]. [1] Diet variety comprised cereals and tubers, animal-based foods, beans and bean products, as well as vegetables and fruits. Consumption of the food subgroups of these four main food types was the basis for calculating the diet variety score for the DQI diet variety component (Table S1). A score of 1 for a food subgroup was provided if more than 25 g of the subgroup was consumed. The main food type score was obtained by calculating the proportion of food subgroups within a main food type of which at least 25 g were consumed. To calculate the diet variety score, we first set an intermediate variable m and then set the weight for the vegetables and fruits score in m to 40% and the weight for the scores of the three other types to 20% each. Therefore, $m = \textit{Cereals and Tubers} \times 20\% + \textit{Animal Foods} \times 20\% + \textit{Beans and dairy products} \times 20\% + \textit{Vegetables and fruit} \times 40\%$. Subsequently, m was divided into 13 groups from the lowest to highest scores. The diet variety scores of the individuals from the lowest scoring group to the highest scoring group ranged from −12 to 0, increasing in increments of 1; [2] The fruits and vegetables score was obtained from the corresponding values derived by calculating the consumption of fruits and vegetables and carotene-rich vegetables (i.e., vegetables rich in carotene). The range of values for the fruits and vegetables score was from −12 to 0. To calculate the fruits and vegetables score, we first set an intermediate

variable n and then recruited individuals with a daily intake of more than 2600 kcal $n = 6\left[\left(TVF/700\right) + \left(CV/300\right)\right]$ and less than or equal to 2600 kcal $n = 6\left[\left(TVF/500\right) + \left(CV/200\right)\right]$, where TVF is total vegetables and fruits consumed (g) and CV is carotene-rich vegetables (g). Subsequently, n was divided into 13 groups from the lowest to highest scores. The fruit and vegetables scores of the individuals from the lowest scoring group to the highest scoring group ranged from −12 to 0, increasing in increments of 1; [3] RNI refers to recommended nutrient intake, and is the nutrient intake level that can satisfy the vast majority of people of a specific gender, age, and physiological condition (97%–98%); provided by China Food Composition Table [2] (Table S3).

Table S2. Diet variety food subgroups.

Food Subgroups	Representative Foods
Cereals and Tubers	
Rice and products	Steamed/boiled rice, rice products
Wheat and products	Bread, pancake, noodles
Corn, coarse grains and products	Cornfour products, barley, oatmeal millet, buckwheat
Starchy roots and products	Sweet potato, yam, taro
Animal Foods	
Red meat and products	Beef, pork, lamb, liver, sausage
Poultry and game	Chicken, duck, rabbit
Egg	Hen egg, duck egg
Fish and shellfsh	Fish, shrimp, mussel
Beans and dairy products	
Legumes and products	Soybean, mung bean, fresh and dried tofu
Milk and dairy products	Milk, milk powder, cheese
Vegetables and fruit	
Dark-colored vegetables	Spinach, carrot, tomato
Light-colored vegetables	Cabbage, potatoes, cucumber, pickles
Fruit	Fresh and dried fruit

Table S3. Chinese RNI for energy and calcium.

Activity Level	Energy (kcal) RNI [1]		Calcium (mg) RNI [1]	
	Male	Female		
Age 20–49				
Light	2400	2100	800	
Moderate	2700	2300	800	
Heavy	3200	2700	800	
Age 50–59				
Light	2300	1900	1000	
Moderate	2600	2000	1000	
Heavy	3100	2200	1000	
Pregnant				
Light	——	2300	0–3 months	800
Moderate	——	2500	4–6 months	1000
Heavy	——	2900	7–10 months	1200
Lactating				
Light	——	2600	1200	
Moderate	——	2800	1200	
Heavy	——	3200	1200	

[1] Source: Institute of Nutrition and Food Safety [2].

Table S4. Mean intake of selected foods and nutrients by DQI total score.

DQI and Components	2004 (*n* = 3083)		2006 (*n* = 3069)		2009 (*n* = 3319)		2011 (*n* = 4362)		*p*	Net Changes [2]
	Mean	SD [1]	Mean	SD [1]	Mean	SD [1]	Mean	SD [1]		
Total DQI score	−9.24	19.31	−7.22	18.65	−13.13	15.88	−13.78	15.00	0.15	−4.54
Cereals and tubers (g)	1359.34	600.96	1263.14	560.04	1264.18	545.51	1058.35	590.02	0.11	−300.99
Fruit and vegetable (g)	1054.67	612.28	1100.88	720.76	1038.03	570.04	872.57	601.52	0.21	−182.10
Animals foods (g)	398.13	365.40	433.17	348.80	446.78	322.85	405.77	305.74	0.78	7.64
Milk and dairy products (g)	28.45	152.78	21.33	101.41	17.33	100.33	52.33	176.83	0.47	23.88
Legumes and products (g)	138.79	197.65	150.86	217.89	160.76	210.68	133.43	204.46	0.97	−5.36
Protein (% energy)	12.17	2.69	12.24	2.63	12.57	2.75	13.82	3.47	0.12	1.65
Calcium (mg)	374.28	255.74	366.09	314.56	336.82	211.69	290.42	312.50	0.05	−83.86
Total energy (kcal)	2266.39	660.11	2240.35	657.97	2201.26	668.51	1989.58	687.90	0.13	−276.81
Total carbohydrate (% energy)	59.29	11.88	57.09	11.97	55.99	10.76	52.06	11.92	0.05	−7.23
Total fat (% energy)	27.60	11.21	29.62	11.27	30.58	10.37	33.31	11.71	0.03	5.71
Saturated fat (% energy)	8.73	185.96	5.95	48.99	0.95	1.30	1.05	1.31	0.04	−7.68
Sodium (mg)	5779.69	5511.40	5529.32	6797.90	672.01	775.18	641.18	682.05	0.06	−5138.51
Alcohol (mL)	3.70	18.01	3.63	17.98	2.79	16.30	2.50	14.67	0.03	−1.20

[1] SD is the abbreviation of standard deviation; [2] Net change between 2011 and 2004.

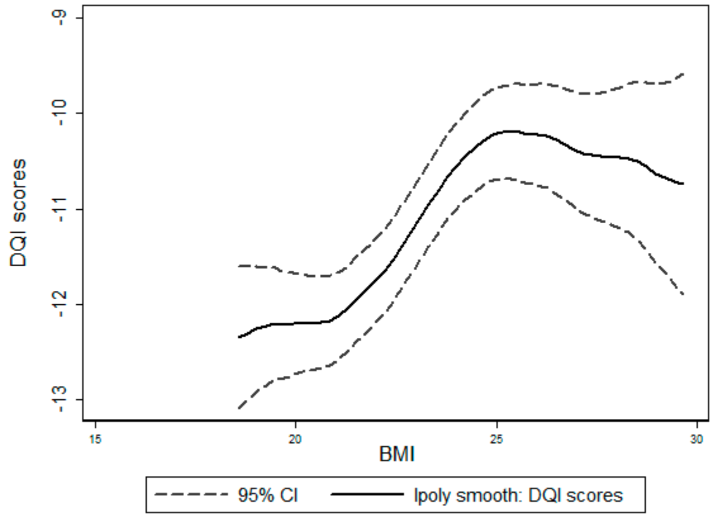

Figure S1. Association between DQI and BMI. 1. Solid line refers to fitted value, and the two dashed lines refer to the 95% confidence interval; 2. Lower and upper 5% values are censored to remove outliers.

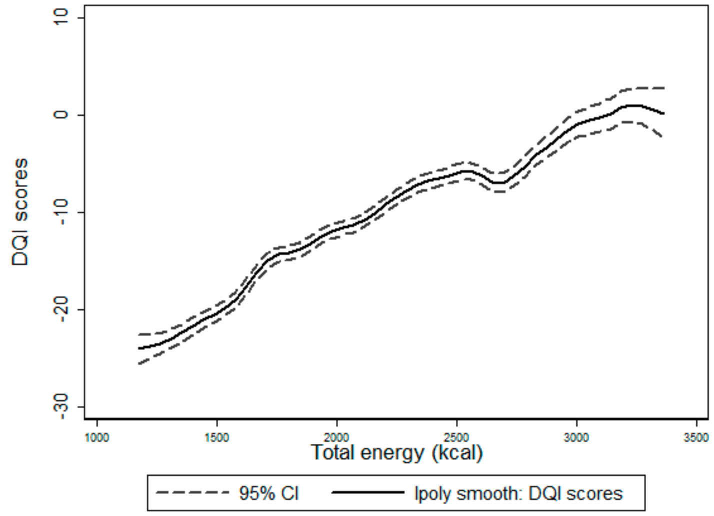

Figure S2. Association between DQI and total energy. 1. Solid line refers to fitted value, and the two dashed lines refer to the 95% confidence interval; 2. Lower and upper 5% values are censored to remove outliers.

References

1. Stookey, J.D.; Wang, Y.; Ge, K.; Lin, H.; Popkin, B.M. Measuring diet quality in China: The INFH-UNC-CH diet quality index. *Eur. J. Clin. Nutr.* **2000**, *54*, 811–821.
2. Institute of Nutrition and Food Safety. *China Food Composition 2009*; Peking University Medical Press: Beijing, China, 2009. (In Chinese)

© 2016 by the authors; licensee MDPI, Basel, Switzerland. This article is an open access article distributed under the terms and conditions of the Creative Commons by Attribution (CC-BY) license (http://creativecommons.org/licenses/by/4.0/).
